# *In vitro* activity, safety and *in vivo* efficacy of the novel bumped kinase inhibitor BKI-1748 in non-pregnant and pregnant mice experimentally infected with *Neospora caninum* tachyzoites and *Toxoplasma gondii* oocysts

**DOI:** 10.1016/j.ijpddr.2021.05.001

**Published:** 2021-05-18

**Authors:** Dennis Imhof, Nicoleta Anghel, Pablo Winzer, Vreni Balmer, Jessica Ramseier, Kai Hänggeli, Ryan Choi, Matthew A. Hulverson, Grant R. Whitman, Samuel L.M. Arnold, Kayode K. Ojo, Wesley C. Van Voorhis, J. Stone Doggett, Luis M. Ortega-Mora, Andrew Hemphill

**Affiliations:** a Institute of Parasitology, Vetsuisse Faculty, University of Bern, Switzerland; b Graduate School for Cellular and Biomedical Sciences (GCB), University of Bern, Switzerland; c Center for Emerging and Re-emerging Infectious Diseases (CERID), Division of Allergy and Infectious Diseases, Department of Medicine, University of Washington School of Medicine, Seattle, WA, USA; d Department of Pharmaceutics, University of Washington, Seattle, WA, USA; e Departments of Global Health and Microbiology, University of Washington, Seattle, WA, USA; f VA Portland Health Care System, Research and Development Service, Portland, OR, USA; g SALUVET, Animal Health Department, Faculty of Veterinary Sciences, Complutense University of Madrid, Ciudad Universitaria s/n, Madrid, Spain

**Keywords:** Bumped kinase inhibitors, Calcium dependent protein kinase inhibitor, Neosporosis, Toxoplasmosis, Pregnancy, Tachyzoites, Oocysts, Treatment

## Abstract

Bumped kinase inhibitors (BKIs) target the apicomplexan calcium-dependent protein kinase 1 (CDPK1). BKI-1748, a 5-aminopyrazole-4-carboxamide compound when added to fibroblast cells concomitantly to the time of infection, inhibited proliferation of apicomplexan parasites at EC_50_s of 165 nM (*Neospora caninum)* and 43 nM (*Toxoplasma gondii)*. Immunofluorescence and electron microscopy showed that addition of 2.5 μM BKI-1748 to infected HFF monolayers transformed parasites into multinucleated schizont-like complexes (MNCs) containing newly formed zoites, which were unable to separate and form infective tachyzoites or undergo egress. In zebrafish (*Danio rerio*) embryo development assays, no embryonic impairment was detected within 96 h at BKI-1748 concentrations up to 10 μM. In pregnant mice, BKI-1748 applied at days 9–13 of pregnancy at a dose of 20 mg/kg/day was safe and no pregnancy interference was observed. The efficacy of BKI-1748 was assessed in standardized pregnant mouse models infected with *N. caninum* (NcSpain-7) tachyzoites or *T. gondii* (TgShSp1) oocysts. In both models, treatments resulted in increased pup survival and profound inhibition of vertical transmission. However, in dams and non-pregnant mice, BKI-1748 treatments resulted in significantly decreased cerebral parasite loads only in *T. gondii* infected mice. In the *T. gondii*-model, ocular infection was detected in 10 out of 12 adult mice of the control group, but only in 3 out of 12 mice in the BKI-1748-treated group. Thus, TgShSp1 oocyst infection is a suitable model to study both cerebral and ocular infection by *T. gondii.* BKI-1748 represents an interesting candidate for follow-up studies on neosporosis and toxoplasmosis in larger animal models.

## Graphical abstract

**Figure:**
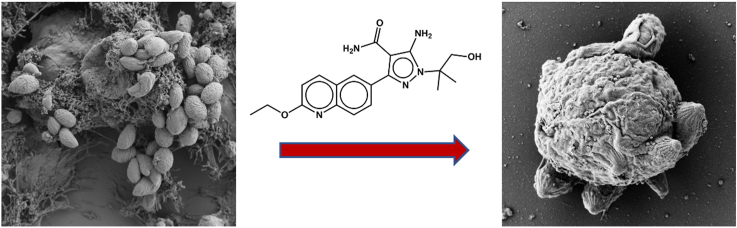

## 1 Introduction

The apicomplexan parasites *Neospora caninum* and *Toxoplasma gondii* cause important diseases in farm animals and have an enormous global economic impact. *N. caninum* is one of the major infectious causes of abortion and stillbirth in cattle (Dubey et al., 2017). *T. gondii* has a higher impact due to infections in a wider range of hosts, including humans (Webster and Dubey, 2010). The two protozoans exhibit life cycles comprised of three distinct stages: (i) sporozoites, encapsulated in oocysts that are formed within their respective definitive hosts (feline for *T. gondii*, canine for *N. caninum*) and which are shed with the feces; (ii) rapidly proliferating tachyzoites causing acute disease that can be vertically transmitted; and (iii) slowly proliferating bradyzoites, which form tissue cysts, and persist for years without clinical signs (Dubey et al., 2017). Intermediate hosts get infected by oral uptake of oocysts or by ingestion of tissue cysts. In an immunocompetent host, conversion to bradyzoites occurs, and cysts are formed mostly within the brain and muscle tissue (Webster and Dubey, 2010; Dubey et al., 2017). At this stage, the parasites do not cause disease. However, problems can occur during pregnancy or immunosuppression. Toxoplasmosis is a major abortion-causing pathogen in sheep and other farm animal, and primary infection in pregnant women with *T. gondii* can lead to vertical transmission, fetal malformations, or abortion (Hampton, 2015). In patients undergoing immunosuppressive disease or therapy, reactivation of bradyzoites and re-differentiation into tachyzoites often cause serious pathology. No cases of human *N. caninum* infection have been reported to date, but neosporosis-induced abortion and disease in cattle and other ruminants is a major economic burden (Aguado-Martínez et al., 2017).

Calcium-dependent protein kinase 1 (CDPK1), represents a promising drug target that is found in numerous apicomplexans. Bumped kinase inhibitors (BKIs) specifically inhibit the activity of CDPK1. The importance of CDPK1 was first demonstrated in *T. gondii* gliding motility, exocytosis, invasion and egress (Kieschnick et al., 2001; Nagamune et al., 2008). Crystal structures of *T. gondii* CDPK1 revealed a glycine gatekeeper residue at the entry of the ATP binding pocket (Murphy et al., 2010; Ojo et al., 2010, 2014; Wernimont et al., 2010). This allows selective targeting of CDPK1 with BKIs, taking advantage of a hydrophobic pocket that is opened up next to the glycine gatekeeper (Bishop et al., 1998; Bishop and Shokat, 1999). Mammalian protein kinases almost uniformly have gatekeeper sidechains that are larger than the hydrogen in glycine and thus are usually not inhibited by BKIs (Keyloun et al., 2014).

Several BKIs have shown promising efficacy against *N. caninum* and *T. gondii in vitro* and *in vivo*. Toxoplasmosis and neosporosis are systemic infections, thus for optimal therapy, these compounds should penetrate the blood-brain barrier, the placenta and the eyes (Arnold et al., 2017). For two pyrazolopyrimidine (PP) BKI compounds, BKI-1294 (Ojo et al., 2014; Winzer et al., 2015; Müller et al., 2017a; Sánchez-Sánchez et al., 2019a) and BKI-1553 (Müller et al., 2017b; Sánchez-Sánchez et al., 2018a), proof of concept has been demonstrated in small and large animal models of toxoplasmosis and neosporosis.

This study reports on another member of the BKI-class, the 5-aminopyrazole-4-carboxamide (AC) compound BKI-1748. BKI-1748 has a number of attractive efficacy and safety features. It inhibits *Tg*CDPK1 and *Nc*CDPK1 enzyme activities at low nM-concentrations and has excellent pharmacokinetics after oral dosing with 25 mg/kg in mice, achieving 38.6 μM maximum concentration with an area-under-the-curve of 11412 min*μmol/L and half-life of 164 min, allowing once daily-dosing (Huang et al., 2019). Solubility is > 100 μM in aqueous solution at pH 6.5 and 2.0, and BKI-1748 is only 80% mouse-plasma-bound (Huang et al., 2019). BKI-1748 exhibited efficacy against acute and chronic *T. gondii* infection with RH- and Prugniaud-strains, respectively (Hulverson et al., 2019), no detectable inhibition at 30 μM of human-ether-à-go-go (hERG) K^+^ -channel, a cardiovascular risk (Huang et al., 2019), no inhibition at 10 μM against SRC, a mammalian protein kinase with a small gatekeeper residue, suggesting little off-target kinase inhibition (Hulverson et al., 2019), and favorable rat and dog cardiovascular safety testing, with only minor abnormalities detected at 18 μM and above (Choi et al., 2020). These features favor further studies on this compound for efficacy in mouse pregnancy against *Toxoplasma* and *Neospora.*

## 2 Material and methods

### 2.1 Cell culture media, biochemicals, BKI-1748

Culture media were from Gibco-BRL (Zürich, Switzerland), biochemicals from Sigma (St. Louis, MO, USA). BKI-1748 was originally synthesized in the Department of Biochemistry of the University of Washington, USA (Hulverson et al., 2019) and scaled up by WuXi Apptec Inc., Wuhan, China to >98% purity by LC/MS-MS and NMR and shipped as powder. For *in vitro* studies, stock solutions of 20 mM were prepared in dimethyl-sulfoxide (DMSO) and stored at −20 °C.

### 2.2 Host cells and parasites

Human foreskin fibroblasts (HFF; PCS-201-010™) and BALB/c dermal fibroblasts (CELLNTEC AG, Bern, Switzerland) were maintained as described (Winzer et al., 2020a). The *N. caninum* Spain-7 isolate (NcSpain-7) and the β-galactosidase expressing strain Nc-β-gal were maintained in HFF (Aguado-Martínez et al., 2016). *T. gondii* Me49 and *T. gondii* β-gal tachyzoites were cultured as previously described (Winzer et al., 2015). *T. gondii* oocysts of the type II isolate TgShSp1 (Sánchez-Sánchez et al., 2019b) were obtained from Complutense University of Madrid, Spain, and stored at 4 °C until use.

### 2.3 Cytotoxicity and anti-*T. gondii*/anti-*N. caninum* efficacy assessments

HFF cytotoxicity and *in vitro* efficacy assessments against Tg-β-gal and Nc-β-gal tachyzoites (EC_50_ determinations) were done as described earlier (Barna et al., 2013; Anghel et al., 2020). EC_50_ values were calculated after the logit-log transformation of relative growth and subsequent regression analysis by use of the corresponding software tool in the Excel software (Microsoft, Seattle, WA).

### 2.4 Microscopy

Immunofluorescence labeling was done as described previously (Winzer et al., 2020b). In short, 2 × 10^4^ HFF were grown on glass coverslips and were infected with 2 × 10^4^ NcSpain-7 or TgMe49 tachyzoites for 2 h at 37 °C/5%CO_2._ The medium was replaced and supplemented with 2.5 μM BKI-1748, or medium without compound. Infected cells were cultured for a maximum of 9 days, with medium plus/minus drug being changed daily. On days 2, 6 and 9, cells were fixed in 3% paraformaldehyde in PBS (pH 7.2) for 10 min, and permeabilized in a 1:1 solution of pre-cooled methanol/acetone at −20 °C. Following rehydration, nonspecific binding sites were blocked in PBS/3% bovine serum albumin (BSA) overnight at 4 °C. The monoclonal mouse anti-NcSAG1 (1:1000), anti-mouse fluorescein-isothiocyanate (FITC) (1:300), polyclonal rabbit anti-IMC1 (1:500), and anti-rabbit tetramethyl-rhodamine-isothiocyanate (TRITC) (1:300) were applied sequentially. Finally, samples were mounted in H-1200 Vectashield mounting medium (Vector Laboratories, Burlingame, CA, USA) containing 4,6-diamidino-2-phenylindole (DAPI).

For transmission-(TEM) and scanning-electron microscopy (SEM), HFF grown in T25 or T125 tissue culture flasks were exposed to 1 × 10^7^ NcSpain-7 tachyzoites in culture medium (4 h, 37 °C/5%CO_2_). Then cultures underwent continuous treatment with 2.5 μM BKI-1748 for 4 days. TEM preparations were carried out as previously described (Winzer et al., 2015). Ultrathin sections were placed onto formvar-carbon-coated grids (PLANO, Wetzlar, Germany), and samples were observed on a Philips CM12 TEM operating at 80 kV. For SEM, MNCs and tachyzoites were separated from host cell debris by Sephadex-G-25-chromatography and were fixed and processed as described (Winzer et al., 2020c). They were placed onto glass coverslips, sputter-coated with gold and inspected on a Zeiss Gemini450 SEM operating at 5 kV.

### 2.5 Ethics statement

Animal experiments were approved by the Animal Welfare Committee of the Canton of Bern (licenses BE101/17 and BE117/2020). Animals were handled in strict accordance with practices to minimize suffering. BALB/c and CD1 mice, 6 weeks of age, were purchased from Charles River (Sulzberg, Germany), were maintained in a common room under controlled temperature and a 14 h/10 h light/dark cycle and were housed in the facility for two weeks for adaptation prior to the experiments.

### 2.6 Pregnancy interference test in BALB/c mice

Twenty-four female BALB/c mice were estrus-synchronized for 3 days (Winzer et al., 2015) and 1 male and 2 females per cage were housed together for 72 h. After mating, males were removed, and female mice were randomly allocated to 4 experimental groups (6 mice/group; 2 females/cage). BKI-1748 was supplied by oral gavage at 50, 20 or 5 mg/kg/day in 100 μl corn oil on day 9–13 post-mating (Winzer et al., 2015; Müller et al., 2017a). The placebo group received 100 μl corn oil for 5 consecutive days. One hour after the final drug application, blood from the tail vein of mice treated with 20 mg/kg and 5 mg/kg BKI-1748 was collected and transferred into heparin-coated tubes. After inverting the tubes carefully, blood samples were centrifuged (10min, 1000×*g*), and blood plasma was collected and stored at −20 °C. Following treatments, mice were observed daily and were weighted every third day. At day 18 post-mating, pregnant females were separated into single cages to rear their offspring. Pregnant mice gave birth on days 20–22. Data on fertility, litter size, neonatal mortality, postnatal mortality and the clinical state of dams and pups were recorded daily. Dams and pups were observed for at least 2 weeks, before they were euthanized in a chamber by isoflurane/CO_2_.

### 2.7 Measurements of BKI-1748 concentration in mouse plasma

The methods for measurement of plasma BKI concentrations have been previously described (Hulverson et al., 2019). Briefly, BKI-1748 was extracted from plasma using acetonitrile/0.1% formic acid with an internal standard and a standard curve was prepared for quantification. All LC-MS/MS analytes were measured with an Acquity ultra performance liquid chromatography (UPLC) system in tandem with a Xevo TQ-S micro mass spectrometer (Waters, Milford, MA, USA).

### 2.8 Evaluation of BKI-1748 treatment effects in BALB/c mice infected with NcSpain-7 tachyzoites

34 female and 17 male BALB/c mice, 8 weeks of age, were used. Mating was done as described under 2.6. Subsequently, females were randomly distributed into 3 groups: BKI-1748, BKI-1748+infection (n = 14); C+, corn oil + infection (n = 14); C-, corn oil alone (n = 6). Three days prior to infection, NcSpain-7 tachyzoites were transferred from HFF to BALB/c fibroblasts and cultured at 37 °C/5% CO_2_ (Aguado-Martínez et al., 2018). On day 7 post-mating, parasites were collected from culture flasks and counted as described (Arranz-Solís et al., 2015), and 10^5^ tachyzoites/100μl/mouse were injected subcutaneously. Group C- was inoculated with BALB/c dermal fibroblasts. At 2 days post-infection (p.i.), treatment with BKI-1748 suspended in corn oil at 20 mg/kg/day for 5 days, or with corn oil alone, was initiated. Pregnant mice were distributed into single cages at day 18 post-mating and non-pregnant mice were maintained in cages of 3–4 animals. Mice were evaluated for clinical signs, litter size, neonatal and postnatal mortality. All mice were euthanized 4 weeks post-partum (p.p.) as described above. Blood and brain samples were collected. IgG1-and IgG2a-levels were assessed by ELISA (Debache et al., 2008, 2009; Aguado-Martínez et al., 2016).

### 2.9 BKI-1748 efficacy assessment in CD1 mice orally infected with TgShSp1 oocysts

29 female and 15 male CD1 mice, 8 weeks of age, were used. Following mating, all females were randomly distributed into 3 experimental groups: BKI-1748, BKI-1748+infection (n = 12); C+, corn oil + infection (n = 12); C-, corn oil alone (n = 5). Infections were carried out with 50 TgShSp1 oocysts at day 7 post-mating, the C- group received PBS only. Treatments and separation of pregnant and non-pregnant mice were done as described above. All mice were monitored clinically for 4 weeks p.p. Following euthanasia, blood, brain and eye samples were collected. Total IgG was measured in *T. gondii* infected mice by ELISA (Sánchez-Sánchez et al., 2019b).

### 2.10 Determination of cerebral parasite load by real-time PCR

Quantification of the cerebral parasite load in non-pregnant mice, dams and pups was done by real-time PCR methods designed specifically for either *N. caninum* (Müller et al., 2002; Aguado-Martínez et al., 2018) or *T.* gondii (Costa et al., 2000). For DNA purification the NucleoSpin DNA RapidLyse Kit (Macherey-Nagel, Oensingen, Switzerland) was used according to standard protocols and DNA concentrations were quantified using the QuantiFluor double-stranded DNA (dsDNA) system (Promega, Madison, WI, USA). Quantitative real-time PCR was performed with the Light Cycler (Roche, Basel, Switzerland), and parasite loads were calculated with a standard curve of DNA samples from 1′000, 100, 10 *N. caninum* or *T. gondii* tachyzoites included in each run.

### 2.11 Statistical analysis

Statistical analysis of cerebral and ocular parasite burdens and antibody titers were compared between groups by the non-parametric Kruskal-Wallis test, followed by Mann-Whitney-U test. The pup mortality along time was compared by plotting survival events at each time point in Kaplan-Meier graphs and survival curves were compared by the Log-rank (Mantel-Cox) test. Statistical analysis was performed using Graphpad Prism version 9.0.2 for MacOSX (GraphPad Software, La Jolla, CA, USA).

## 3 Results

### 3.1 In vitro activities of BKI-1748

**Fig. 1:**
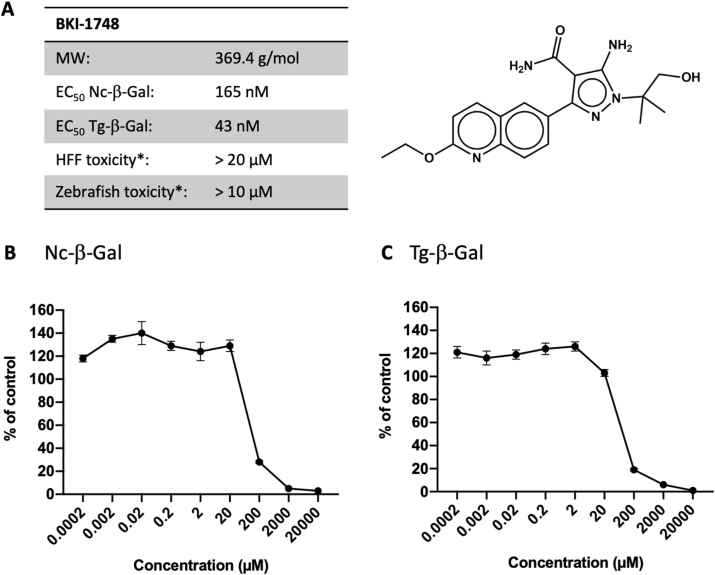
Structure and *in vitro* activities of BKI-1748 against *N. caninum* (Nc-β-gal) and *T. gondii* (Tg-β-gal). (A) molecular structure of BKI-1748, MW = molecular weight; EC_50_ = concentration of half maximal proliferation inhibition; *toxicity levels against human foreskin fibroblasts (HFF) and zebrafish embryos were determined previously [22]. (B,C) Dose-response curves for *Neospora* and *Toxoplasma* tachyzoites grown in HFF.

Different compound concentrations were added to HFF concomitantly to infection with Nc-β-gal or Tg-β-gal tachyzoites. The EC_50_ was 165 nM for *N. caninum* and 43 nM for *T. gondii* (Fig. 1). In agreement with previous studies (Anghel et al., 2020), the viability of uninfected HFF was not impaired by BKI-1748 up to a concentration of 20 μM.

**Fig. 2:**
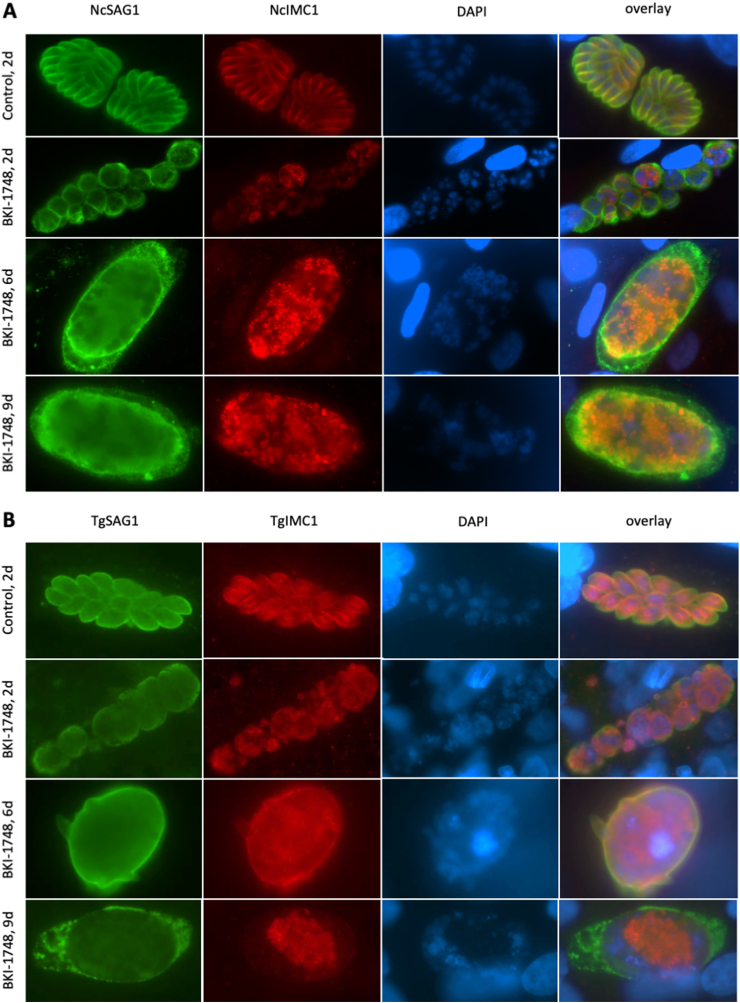
Immunofluorescence staining of HFF monolayers infected with *N. caninum* NcSpain-7 (A) and *T. gondii* Me49 tachyzoites (B) after treatment with 2.5 μM BKI-1748 for 2, 6 and 9 days. As a control, HFF were infected with the respective parasites but not treated and were fixed and stained after 2 days of incubation at 37 °C/5% CO_2_. SAG1 is labelled in green, anti-IMC1 staining is red, and nuclei are stained blue with DAPI. (For interpretation of the references to colour in this figure legend, the reader is referred to the Web version of this article.)

Immunofluorescence staining of *N. caninum* (Fig. 2A) or *T. gondii* infected HFF (Fig. 2B), demonstrated that BKI-1748 treatment did not just inhibit proliferation of tachyzoites but induced the formation of intracellular multinucleated complexes (MNCs), similar to what has been observed earlier with BKI-1294 (Winzer et al., 2020b). These MNCs were enclosed by the major surface antigen SAG1 and contained zoites that underwent active proliferation, as evidenced by labeling with an antibody directed against the inner membrane complex 1 (IMC1).

**Fig. 3:**
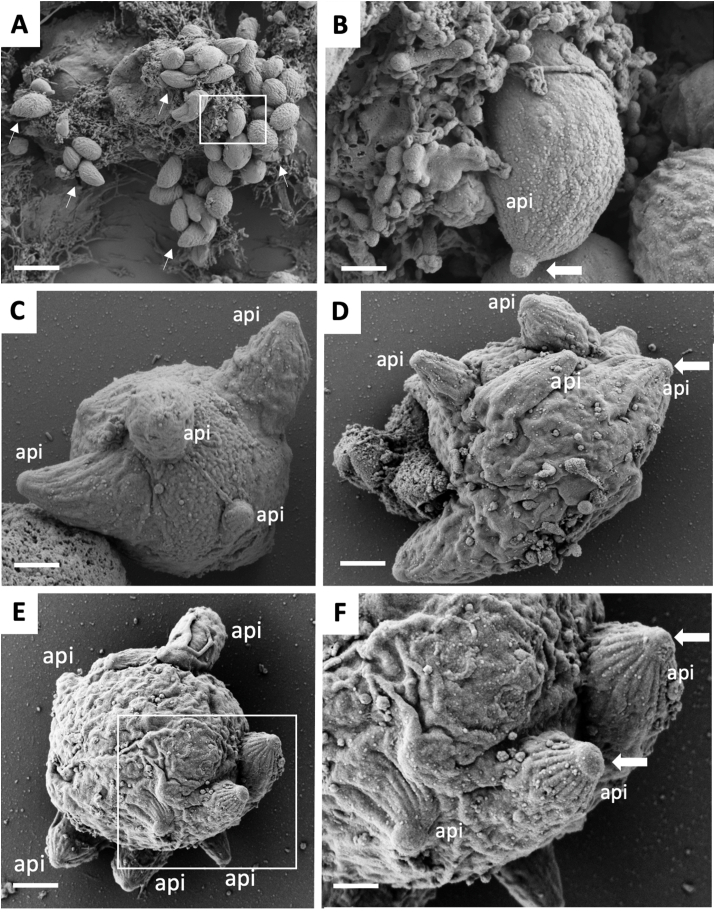
SEM micrographs of *N. caninum* tachyzoites and MNCs after exposure of infected HFF with BKI-1748. (A,B) non-treated tachyzoites, (C–F) BKI-1748 treated MNCs. The framed area in (A) is enlarged in (B), and the framed area in (E) is shown at higher magnification in (F); api = apical complex, small arrows point towards tachyzoites, the horizontal arrows indicate the conoid. Bars in (A) = 2.4 μM; (B) = 0.35 μm; (C) = 0.45 μm; (D) = 0.6 μm; (E) = 0.9 μm; (F) = 0.35 μM.

Fractions that were enriched for *N. caninum* MNCs were prepared, fixed and processed for SEM. MNCs were 2–6 μm in diameter and were comprised of a large parasitic mass with multiple protruding apical complexes displaying a conoid (Fig. 3).

**Fig. 4:**
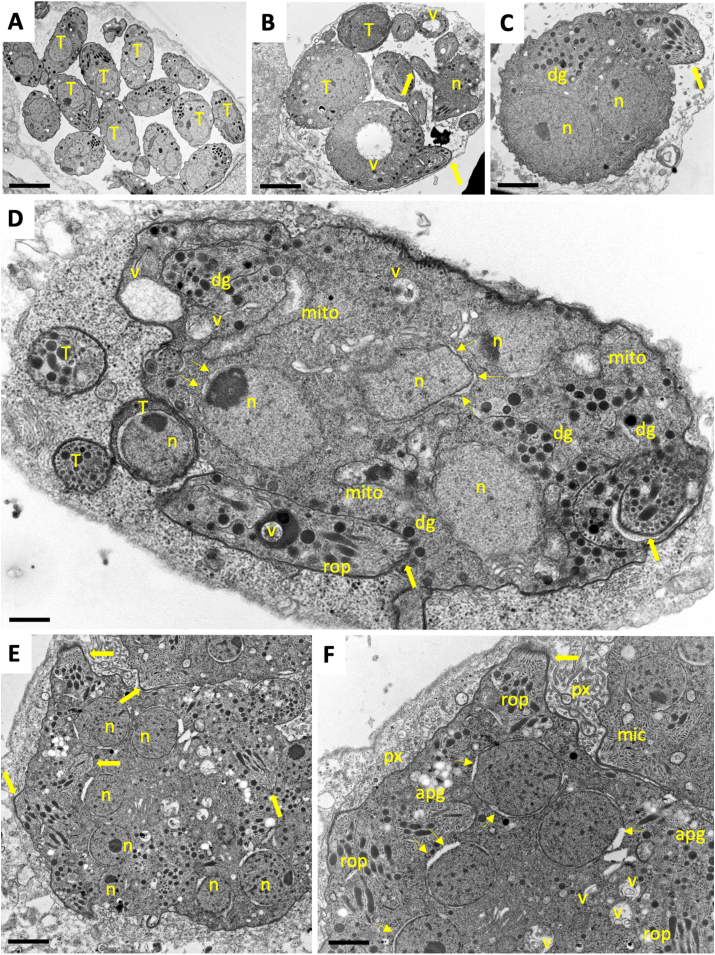
Transmission electron microscopy of *N. caninum* tachyzoites and MNCs. TEM micrographs of tachyzoites within a parasitophorous vacuole (A), and MNCs after 24 h (B,C), 48 h (D) and 72 h of exposure to BKI-1748 (E,F). (F) is a higher magnification view of (E); T indicates individual tachyzoites in BKI-treated cultures; n = nucleus; v = cytoplasmic vacuole; large arrows point towards newly formed apical complexes, dg = dense granules; rop = rhoptries; mito = mitochondrion; mic = micronemes; px = parasitophorous vacuole matrix; apg = amylopectin granules; small arrows indicate separation between nuclear membrane and the cytoplasm. Bars in (A) = 1.2 μm; (B) = 0.8 μm; (C) = 0.6 μm; (D) = 0.25 μm; (E) = 0.5 μm; (F) = 0.3 μm.

**Fig. 5:**
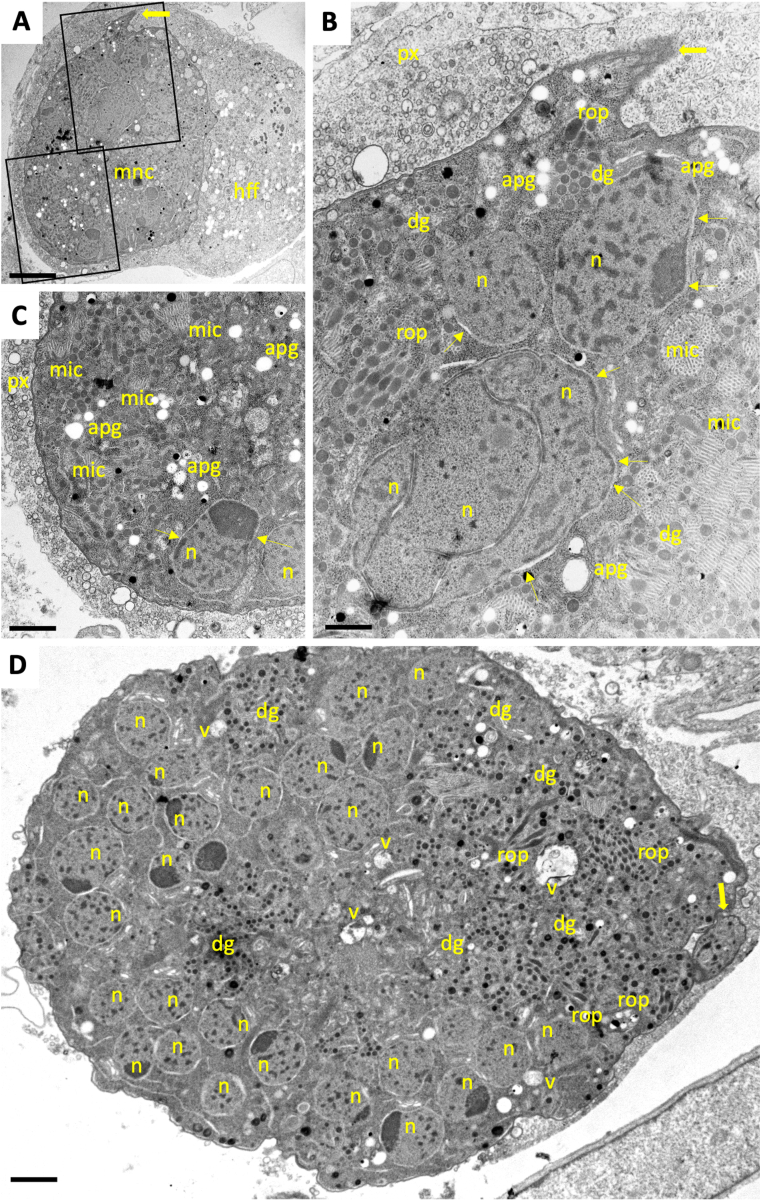
TEM micrographs of *N. caninum* MNCs after 96 h treatment with BKI-1748. (A) is a low magnification view of an MNC infected human foreskin fibroblast, the framed areas are magnified in (B) and (C). (B) shows a cluster of nuclei, surrounded by secretory organelles and amylopectin granules, and micronemes are also arranged in tightly packed clusters: n = nucleus; v = cytoplasmic vacuole, large arrows point towards apical complexes, dg = dense granules; rop = rhoptries; mito = mitochondrion; mic = micronemes; px = parasitophorous vacuole matrix; apg = amylopectin granules; small arrows indicate separation between nuclear membrane and the cytoplasm. Scale bars in (A) = 2 μm; (B) = 0.5 μm; (C) = 0.75 μm; (D) = 0.6 μm.

TEM confirmed the presence of viable zoites within these MNCs. Zoites exhibited an apical complex and the typical secretory organelles such as micronemes, rhoptries and dense granules (Fig. 4). After 24 h of treatment, there were still HFF harboring numerous individual tachyzoites, indicating that parasite proliferation was taking place initially, but some parasites exhibited structural alterations such as increased mass, more than one nucleus, vacuolization, and few newly formed apical complexes (Fig. 4B and C). After 48 h, MNCs showed increased numbers of nuclei (Fig. 4D), mitochondria lost their distinct electron dense matrix, and dissolving cristae became evident. Many of the nuclear membranes were physically separated from the cytoplasm. However, individual parasites were still visible in some cells (Fig. 4D). After 72 h, larger complexes filled with newly emerged apical complexes were found, and the aberrant spacing between nuclei and cytoplasm was more evident (Fig. 4E and F). Nuclei were clustered centrally, and dense granules, micronemes and rhoptries were found more frequently in the periphery. In addition, the cytoplasm exhibited vesicles filled with electron-dense material of unknown nature, and the matrix of the parasitophorous vacuole contained granular and filamentous structures. After 96 h (Fig. 5), complexes became massive and occupied a large part of the host cell cytoplasm. These larger MNCs also contained amylopectin granules. Dense granules were among the most abundant organelles, and the matrix of the parasitophorous vacuole was packed with vesicular and granular material (Fig. 5A–C). As shown in Fig. 5D, larger MNCs exhibited a polarized distribution of newly formed nuclei on one side, and typical apicomplexan secretory organelles on the other side.

### 3.2 Safety during pregnancy in mice

In order to investigate whether BKI-1748 interferes in pregnancy outcome, BALB/c mice were mated and received daily oral applications of BKI-1748 emulsified in corn oil at dosages of 50 mg/kg, 20 mg/kg or 5 mg/kg on days 9–13 post-mating. In the group that received 50 mg/kg, only five pups were born alive, and one died within 24 h after birth, documenting a negative influence of pregnancy outcome (Anghel et al., 2020). In contrast, in the groups treated with 20 mg/kg/day or 5 mg/kg/day, no interference in pregnancy compared to the control group was noted (Table 1).

**Table 1:** Potential pregnancy interference of BKI-1748 in embryo development.

Treatment	Number of pregnant mice	Number of non-pregnant mice	Litter size	Neonatal mortality	Postnatal mortality
5 mg/kg BKI-1748	3/6	3/6	14	0/14	0/14
20 mg/kg BKI-1748	4/6	2/6	20	0/20	0/20
50 mg/kg BKI-1748	3/6	3/6	5	1/5	0/4
Corn oil	3/6	3/6	23	1/23	0/22

**Fig. 6:**
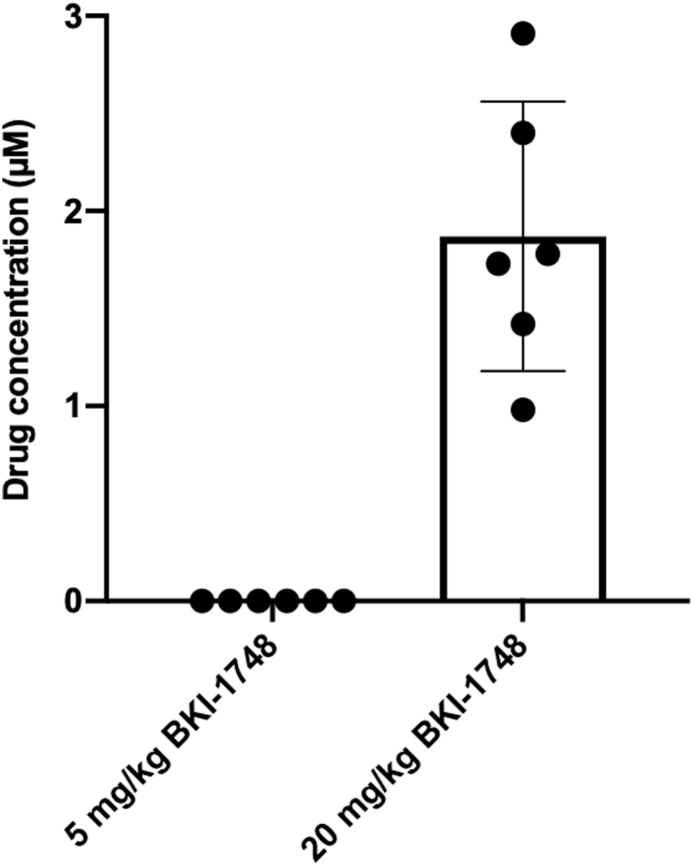
BKI-1748 concentrations measured 1 h after the final treatment at day 5 of 5 mg/kg/day and 20 mg/kg/day. For each dosage, blood of six mice was retrieved through the tail vein and blood plasma was collected to measure the drug concentration by LC-MS/MS.

Drug exposure at the two lower doses was further evaluated by determination of plasma drug concentrations (Fig. 6). Plasma concentrations were below the level of detection in the 5 mg/kg/day group, but mice treated with 20 mg/kg reached concentrations of 0.9–2.9 μM (Fig. 6). These levels were 6–17.5 times higher than the EC_50_ for *N. caninum* and 23–67.4 times higher than the EC_50_ for *T. gondii*.

### 3.3 Efficacy of BKI-1748 in BALB/c mice infected with *N. caninum* tachyzoites

**Fig. 7:**
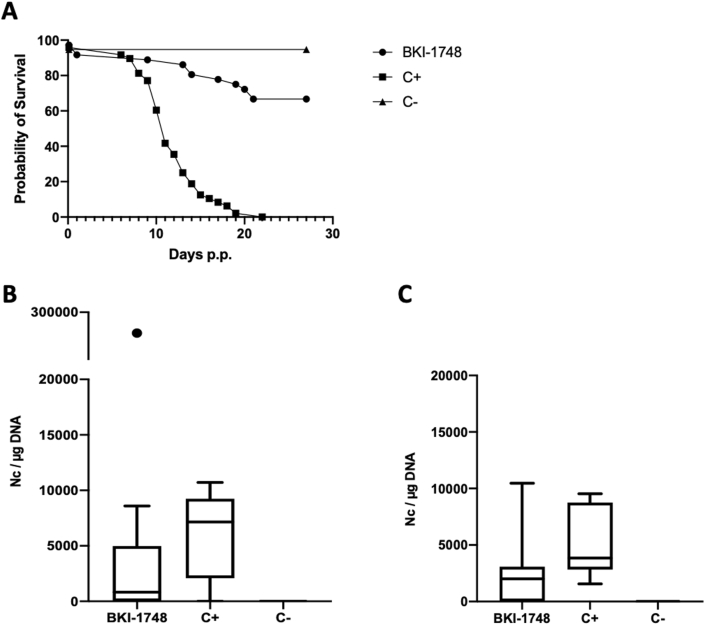
BKI-1748 treatment in the pregnant neosporosis mouse model. (A) Survival curve of pups and cerebral parasite burden of non-pregnant mice (B) and dams (C). Survival rates at each time point were plotted in Kaplan-Meier graphs and curves were compared by the Log-rank (Mantel-Cox) test. Differences between BKI-1748 and positive control curves were highly significant (*P < 0.0001*). BALB/c mice were experimentally infected with 10^5^ NcSpain-7 tachyzoites and treated with BKI-1748 for 5 days at 20 mg/kg/day in corn oil or were treated with corn oil alone (C+). C- was not infected and treated with corn oil only. Brains of all mice were collected directly after euthanasia and parasite burden of non-pregnant mice and dams was quantified by real-time PCR. Values are shown as box plots. No statistically significant differences in the cerebral parasite burden were observed between BKI-1748-treated mice compared to the C+ group (neither in non-pregnant mice nor in dams).

The results of this experiment are summarized in Table 2. None of the dams exhibited clinical signs of neosporosis. Reproductive parameters such as fertility rate or litter size were not altered by the treatment. However, BKI-1748 had a profoundly favorable impact on pup survival (Fig. 7A). Neonatal pup mortality was observed in 2 out of 36 pups born in the BKI-1748 group and 2 out of 48 pups in the C- group, but postnatal mortality was 100% in the C+ group, while only 29.4% (10 out of 34 pups) in the BKI-1748 group (Table 2). ELISA showed that all adult mice had undergone seroconversion. In pregnant animals, IgG1 and IgG2a antibody titers were significantly reduced in the BKI-1748 group compared to the C+ group, while in non-pregnant mice only IgG1 antibody levels were significantly lower (Suppl. file 1).

**Table 2:** Litter size, parasite burden, neonatal and postnatal mortality rates of *N. caninum* infected mice treated with BKI-1748.

Treatment	Challenge	Seropositive for *N. caninum*	*N. caninum* brain positive non-pregnant mice	*N. caninum* brain positive dams	Number of pups per dam	Neonatal mortality	Postnatal mortality	*N. caninum* brain positive pups
20 mg/kg BKI-1748	10^5^ NcSp7 tachyzoites	14/14	4/7	5/7	36/7	2/36	10/34	14/34
Corn oil	10^5^ NcSp7 tachyzoites	14/14	5/6	8/8	48/8	2/48	46/46	46/46
Corn oil	BALB/c dermal fibroblasts	0/6	0/2	0/4	19/4	1/19	0/18	0/18

5 of 7 brain samples of the BKI-1748-treated dams and all 8 brains in the C+ group were *Neospora* PCR-positive (Table 2). The parasite loads in the C+ group were similar to the BKI-1748 group in pregnant and non-pregnant mice (Fig. 7B and C). Vertical transmission in the BKI-1748 treated group was detected in 41% of pups, while all pups tested positive in the C+ group. In non-pregnant mice, *N. caninum* DNA was detected in 4 out of 7 animals in the BKI-1748 group, and in 5 out of 6 mice in the C+ group (Table 2). Thus, BKI-1748 treatment did not inhibit brain infection in adult mice, but significantly inhibited vertical transmission of *N. caninum.*

### 3.4 Efficacy of BKI-1748 treatment in CD1 mice infected with *T. gondii* oocysts

**Fig. 8:**
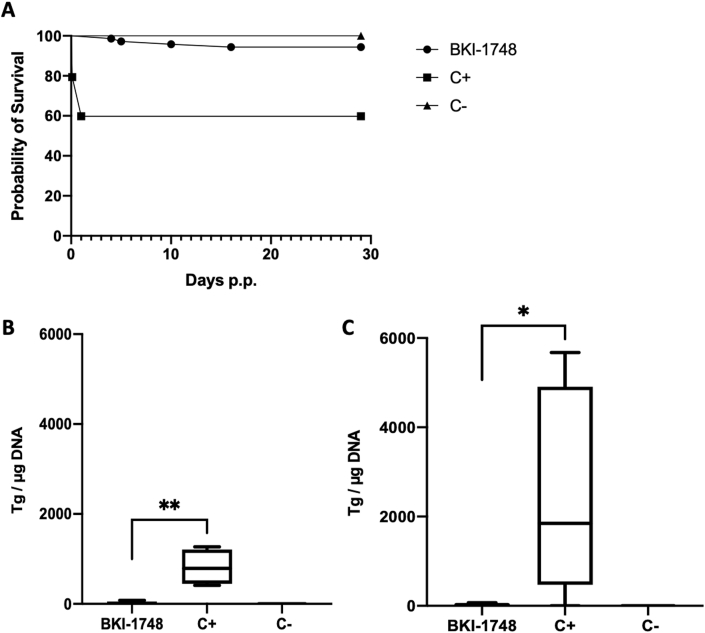
BKI-1748 treatment in the pregnant toxoplasmosis mouse model. (A) Survival curves of pups and cerebral parasite burden of non-pregnant mice (B) and dams (C). Survival rates at each time point were plotted in Kaplan-Meier graphs and curves were compared by the Log-rank (Mantel-Cox) test. Differences between BKI-1748 and positive control curves were highly significant (*P < 0.0001*). CD1 mice were infected with 50 oocysts of the TgShSp1 strain and treated with 20 mg/kg BKI-1748, while C+ was infected and treated with corn oil alone. C- was not infected and treated with corn oil only. All mice were euthanized 30 days p.p. After euthanasia, brains were aseptically collected, and cerebral parasite burden of non-pregnant mice and dams was quantified by real-time PCR. Values are depicted as box plots. Parasite burden between groups were compared by the Kruskal-Wallis test, followed by the Mann-Whitney-U test. Differences between BKI-1748-treated and non-treated groups were statistically significant (*P < 0.0127, **P < 0.0095).

The results of this experiment are summarized in Table 3. Oocyst infection did not result in clinical signs, and reproductive parameters were not affected. A major difference was seen in neonatal mortality (within 2 days p.p.), which was zero in the BKI-1748 group and 40% (37 out of 92 pups) in the C+ group. Four pups in the BKI-1748 treatment group died during the first 16 days of the 1-month follow-up phase, while no postnatal mortality was noted for the C+ group (Fig. 8A). Increased IgG levels were detected in all infected mice, and IgG titers of BKI-1748 treated mice compared to C+ group animals were similar. (Suppl. file 2). In the C+ group 55 pups survived, of which 35 (64%) were PCR-positive. In the BKI-1748 group 67 pups survived, all of which were PCR negative. In the adult mice, brain infection was noted in 7 of 12 BKI-1748 treated mice and in 11 of 12 C+ group mice (Table 3). However, quantification of the parasite load showed highly significant differences between the respective BKI-1748 and C+ groups (Fig. 8B and C).

**Table 3:** Litter size, parasite burden, neonatal and postnatal mortality rates of *T. gondii* infected mice treated with BKI-1748.

Treatment	Challenge	Seropositive for *T. gondii*	*T. gondii* brain positive non-pregnant mice	*T. gondii* brain positive dams	Number of pups per dam	Neonatal mortality	Postnatal mortality	*T. gondii* brain positive pups
20 mg/kg BKI-1748	50 TgShSp1 oocysts	8/12	3/6	4/6	71/6	0/71	4/71	4/71
Corn oil	50 TgShSp1 oocysts	9/12	4/4	7/8	92/8	37/92	0/55	35/55
Corn oil	PBS	0/5	0/2	0/3	41/3	0/41	0/41	0/41

**Fig. 9:**
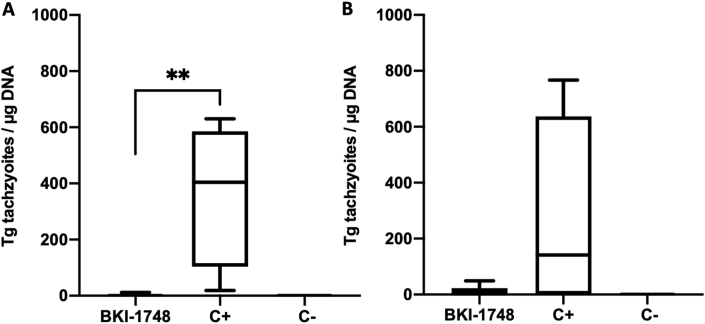
Ocular parasite burden of non-pregnant mice (A) and dams (B) infected with 50 oocysts of the TgShSp1 strain and treated with BKI-1748 at 20 mg/kg/day for 5 days, or with corn oil alone (C+). C- was not infected and treated with corn oil only. Eyes were removed after euthanasia and DNA was extracted and quantified by real-time PCR. Values are shown in box plots. Ocular parasite burden between groups were compared by the Kruskal-Wallis test, followed by the Mann-Whitney-U test. Differences between BKI-1748-treated and non-treated groups were statistically significant in non-pregnant mice (**P < 0.0048) but not in dams.

*Toxoplasma* DNA was also quantified in eye samples from all adult mice. Ocular infection was identified in 2 out of 6 dams in the BKI-1748 group and in 5 out of 8 dams in the C+ group. In non-pregnant mice, ocular infection was strongly reduced in BKI-1748 treated non-pregnant mice contrary to the positive control, with 4 out of 4 mice exhibiting ocular infection (Fig. 9A). *Toxoplasma* eye infection in dams of the BKI-1748 group was not significantly reduced compared to the C+ group (Fig. 9B).

## 4 Discussion

This study describes the efficacy of the CDPK1 inhibitor BKI-1748 against *N. caninum* and *T. gondii in vitro* and in experimentally infected mice. Our results indicate that BKI-1748 represents a promising addition to the growing arsenal of novel candidate compounds that could be developed for the treatment of congenital infection and disease inflicted by these two closely related parasites (Sánchez-Sánchez et al., 2018b; Smith et al., 2021).

*In vitro*, BKI-1748 inhibited both *N. caninum and T. gondii* tachyzoite proliferation at nanomolar values. The *T. gondii* EC_50_ value (43 nM) was 4-times lower compared to the EC_50_ measured for *N. caninum* (165 nM). BKI-1748 did not affect HFF at a concentration up to 20 μM, and did not induce cytotoxicity in human hepatocyte (HepG2) and lymphocyte (CRL-8155) cell lines (Anghel et al., 2020). Microscopy demonstrated that *in vitro* treatment with BKI-1748 at 2.5 μM did not rapidly kill tachyzoites, but, as seen earlier for other BKIs (Winzer et al., 2015, 2020b; Müller et al., 2017b), exposure to BKI-1748 resulted in the appearance of MNCs, which grew considerably with increasing duration of treatment. DNA replication and nuclear division obviously took place in these complexes, but the final steps in cytokinesis were blocked, and newly formed zoites, lacking the major tachyzoite surface antigen SAG1 but expressing IMC1, were trapped within their host cell. While these zoites exhibited some structural alterations, they remained largely viable upon continuous treatment for several days to weeks.

Multinucleated forms were also reported upon BKI-treatment of the related apicomplexans *Sarcocystis neurona* and *Besnoitia besnoiti* (Ojo et al., 2017; Jiménez-Meléndez et al., 2017). A detailed study on *N. caninum* MNCs that formed upon treatment with the related PP compound BKI-1294 showed that upon drug removal parasites resumed their cell cycle and formed infective tachyzoites that re-emerged out of these complexes within 10 days (Winzer et al., 2020b). Overall, MNCs are regarded as a drug-induced stage that ensures survival of the parasite under drug pressure, and comparative proteomics had shown that only 12 proteins were expressed at higher levels in MNCs compared to tachyzoites (Winzer et al., 2020c). Among them were the bradyzoite antigens NcSAG4 and NcBSR4, and other proteins comprised of signal peptides and SRS domains. In addition, mRNA coding for NcBAG1, another bradyzoite marker, was highly expressed in MNCs but not in tachyzoites (Winzer et al., 2015, 2020c). There is evidence that the formation of MNCs also affects the cellular and humoral immune response and could have an impact in terms of re-infection during pregnancy in previously infected animals (Winzer et al., 2020a). Whether MNCs induced by the AC compound BKI-1748 exhibit a similar pattern of upregulated proteins has not been investigated so far.

It was shown previously that exposure of zebrafish embryos to BKI-1748 at concentrations of 10 μM or below did not interfere directly in embryo development (Anghel et al., 2020). This information is important for drugs to be applied during pregnancy. BKI-1748 clearly interfered in pregnancy outcome when applied at 50 mg/kg from days 9–13 of pregnancy (Anghel et al., 2020), a regimen that had been demonstrated to be safe for BKI-1294 treatments (Winzer et al., 2015). However, dosages of 20 and 5 mg/kg/day, did not lead to adverse effects in dams or pups. Determination of BKI-1748 plasma levels in mice treated with 20 mg/kg/day (0.9–2.9 μM) were well below the 10 μM that could potentially exert embryotoxicity. Thus, a dosage of 20 mg/kg/day for 5 days was chosen for treatment studies in the neosporosis and toxoplasmosis mouse models.

In both models, BKI-1748 was highly efficacious in preventing vertical transmission and clinical signs in pups. BKI-1748 appeared more efficacious in the *Toxoplasma* model compared to the *Neospora* model. However, direct comparisons should not be made. *T. gondii* infection was performed in outbred CD1 mice by oral gavage of 50 oocysts of the TgShSp1 strain (Sánchez-Sánchez et al., 2019b). This infection dose had been previously determined in a small pilot experiment not to cause clinical signs in dams but to still provide sufficient vertical transmission (data not shown). On the other hand, 10^5^ *N. caninum* tachyzoites were used to infect inbred BALB/c mice by subcutaneous injection. In the former, a relatively small number (approx. 400) of infective sporozoites were inoculated, which required some time to disseminate and differentiate into the proliferative form that causes disease; in the latter, a much higher number of infective tachyzoites was likely to disseminate much more rapidly, leading to a higher parasite burden within a shorter time frame.

Overall, treatment with BKI-1748 in the neosporosis model was less efficacious compared to the previously reported studies on BKI-1294 (Winzer et al., 2015, 2020a), but clearly superior in terms of safety and efficacy compared to BKI-1553 and BKI-1517 (Müller et al., 2017b), respectively. In contrast to the neosporosis model, the cerebral parasite loads of *Toxoplasma* infected adult mice in BKI-1748-treated versus C+ groups were significantly reduced. This difference in efficacy in adult mice could also be related to the inherent differences in the life cycle stages, the infection dose, and the route of infection.

With regard to the toxoplasmosis mouse model used here, BKI-1748 appeared to exhibit some advantages compared to BKI-1294. BKI-1294 had been assessed in a *T. gondii* Me49 oocyst infection model. In that study, the number of BKI-1294-treated CD1 dams that gave birth to offspring was reduced by 50% compared to the control, which implied a negative impact on fertility in that particular mouse strain, posing some potential safety issues (Müller et al., 2017a). In terms of vertical transmission, however, BKI-1294 also conferred a similarly high degree of protection. However, the PP-compound BKI-1294 was found to exhibit a high hERG liability, which would preclude its use in humans due to safety issues (Choi et al., 2020). BKI-1748 is structurally based on an AC scaffold. AC scaffold compounds were shown to elicit lower affinity for hERG compared to BKIs with a PP scaffold (Choi et al., 2020), rendering this compound more safe and more promising.

In conclusion, the novel CDPK1 inhibitor BKI-1748 is an auspicious compound with proven *in vitro* activity and *in vivo* efficacy against both apicomplexan parasites *N. caninum* and *T. gondii*. Based on these results, BKI-1748 should be considered as a candidate for further assessments in pregnant neosporosis and toxoplasmosis ruminant models.

## Funding

This study was financed by the 10.13039/100000001Swiss National Science Foundation (SNSF) grant 310030_184662, the 10.13039/100000002National Institutes of Health (NIH) grants R01AI089441, R01AI111341, R01HD080670, R01AI155412, R01HD102487, R21AI123690, and R21AI140881, and 10.13039/100000199United States Department of Agriculture, 10.13039/100005825National Institute of Food and Agriculture grants # 2019-07512 and # 2014-06183.

## Declaration of competing interest

WCVV is an owner/officer of ParaTheraTech Inc, a company which is seeking to bring bumped kinase inhibitors to the animal health market.
