# Supplementary material for: In vitro activity, safety and in vivo efficacy of the novel bumped kinase inhibitor BKI-1748 in non-pregnant and pregnant mice experimentally infected with Neospora caninum tachyzoites and Toxoplasma gondii oocysts
Source: Int J Parasitol Drugs Drug Resist. 2021 May 18;16:90–101. doi: 10.1016/j.ijpddr.2021.05.001 (PMC8144743; doi:10.1016/j.ijpddr.2021.05.001)
Supplement: Multimedia component 1 [file mmc1.docx]

**Supplementary files**

**Supplementary file 1:** *N. caninum* IgG1 and IgG2a antibody titers measured from serum collected at the end of the experiment (42 days after challenge). Results are expressed as the mean of RIPC (relative index per cent) compared to the positive control (C+). ** P ≤ 0.0059, *** P ≤ 0.0003, ns = not significant (Mann-Whitney-U test).

**Supplementary file 2:** *T. gondii* IgG antibody titers measured from serum collected at the end of the experiment (42 days after challenge). Results are expressed as the mean of RIPC (relative index per cent) compared to the positive control (C+). No significant differences were observed between IgG antibody titers of BKI-1748 treated non-pregnant mice and dams compared to C+. ns = not significant (Mann-Whitney-U test).
